# Effects of exercise initiation and smoking cessation after new-onset type 2 diabetes mellitus on risk of mortality and cardiovascular outcomes

**DOI:** 10.1038/s41598-022-14603-1

**Published:** 2022-06-23

**Authors:** Mee Kyoung Kim, Kyungdo Han, Bongsung Kim, Jinyoung Kim, Hyuk-Sang Kwon

**Affiliations:** 1 Division of Endocrinology and Metabolism, Department of Internal Medicine, Yeouido St. Mary’s Hospital, College of Medicine, The Catholic University of Korea, #10 63-ro, Yeongdeungpo-gu, Seoul, 07345 South Korea; 2 Department of Statistics and Actuarial Science, Soongsil University, Seoul, 07040 Korea

**Keywords:** Diabetes complications, Lifestyle modification

## Abstract

Lifestyle changes after a diagnosis of type 2 diabetes mellitus (DM) can affect vascular health outcomes. The objective of this study was to investigate the effects of changes in smoking and exercise on the risk of cardiovascular disease (CVD) and mortality in patients with newly diagnosed DM. Data were analyzed for 181,591 people with newly diagnosed type 2 DM who underwent 2 serial health examinations within 2 years before and after DM diagnosis. The study population was followed from the baseline to the date of death or cardiovascular events, or until December 31, 2018 and median follow-up was 6.07 years. Based on the change in status from before to after the diagnosis, participants were grouped into smoking groups (continuous smokers, quitters, new smokers, and nonsmokers) and exercise groups (constant exercisers, new exercisers, exercise dropouts, and nonexercisers). Compared with the nonexercisers, those who initiated exercise after their DM diagnosis had a lower risk of myocardial infarction (MI), stroke, and all-cause mortality: the hazard ratio (HR; 95% confidence interval [CI]) was 0.85 (0.76–0.94) for MI, 0.86 (0.78–0.94) for stroke, and 0.84 (0.89–0.90) for all-cause mortality. Quitters had a higher risk of MI, stroke, and all-cause mortality than nonsmokers, but their risk level was much lower than that in continuous smokers. When the group of continuous smokers and nonexercisers was considered as the reference group, participants who quit smoking and remained nonexercisers had a 21% lower risk of CVD (HR 0.79; 95% CI 0.70–0.90). Those who quit smoking and started exercising had a 46% reduced risk of CVD (HR 0.54; 95% CI 0.41–0.71) and a 22% reduced risk in all-cause mortality (HR 0.78; 95% CI 0.63–0.96). Smoking cessation and exercise initiation after a diagnosis of new-onset type 2 DM was associated with a reduced risk of CVD and all-cause mortality.

## Introduction

The identification and management of modifiable risk factors are crucial for improving outcomes in patients with type 2 diabetes mellitus (DM). Although new antidiabetic drugs have shown significant efficacy in controlling hyperglycemia and reducing incidence of atherosclerotic cardiovascular disease (CVD), lifestyle management still serves a pivotal role in diabetes care^1–3^. Previous research has shown that smoking and physical inactivity are associated with cardiovascular complications related to atherosclerotic CVD in people with type 2 DM^4^. In patients with newly diagnosed DM, smoking cessation combined with weight loss was associated with a significant increase in the risk of mortality, which was higher than that in sustained smokers^4^. The risk of mortality decreased nonsignificantly in patients who quit smoking (“quitters”) but gained body weight, and their risk of mortality was similar to that in sustained smokers^4^. Although smoking cessation appears to reduce the risk of adverse health outcomes over the long term, few studies have estimated the potential reduction in the risk of mortality or CVD from smoking cessation in patients after a diagnosis of type 2 DM^5–7^.

The effects of exercise on the risk of CVD or mortality seem to vary depending on age, comorbidities, and the severity of underlying diseases. Evidence from randomized control trials (RCTs) suggest that exercise does not reduce all-cause mortality or incident CVD in older adults or in people with chronic conditions including DM^8,9^. Although exercise has long been considered a cornerstone in the management of type 2 DM, the epidemiologic data related to the association between the initiation of exercise and cardiovascular complications among patients with newly diagnosed type 2 DM are limited.

There is limited evidence of the effects of lifestyle changes, such as exercise or smoking habits, on the long-term clinical outcomes after a diagnosis of DM. We investigated the effects of changes in smoking and exercise on the risk of CVD and all-cause or cardiovascular mortality in patients with newly diagnosed DM. We also investigated the combined effects of smoking cessation and initiation of exercise on health outcomes after a DM diagnosis.

## Methods

### Study population

In this study, we used the Korean National Health Insurance Service (NHIS) database, which is the national claims database linked with the National Health Screening Program database. In Korea, the NHIS is the single insurer, which is managed by the government and includes all Koreans. Enrollees in the National Health Insurance Corporation are recommended to undergo a standardized medical examination every 2 years^10–12^. The Korean National Health Screening Program employed a standardized questionnaire for smoking status and exercise. Numerous previous reports have used the Korean NHIS Database (DB)-National Health Check-up DB to conduct epidemiologic studies, and its validity has been demonstrated elsewhere^10–14^. From the NHIS database, we identified 1,133,084 patients newly diagnosed with type 2 DM between 2009 and 2012. Patients who did not undergo a follow-up health screening examination within 2 years (n = 915,224) were excluded, as were those with a previous history of myocardial infarction (MI) or stroke (n = 27,966) and those with missing data (n = 8303). Finally, the study population included 181,591 people. Type 2 DM was defined according to the International Classification of Disease (10th Revision [ICD-10]) codes E11–14 for type 2 DM as either the principal diagnosis or the first to fourth additional diagnoses, and the prescription of ≥ 1 antidiabetic drug in a given year.

All procedures performed in studies involving human participants were in accordance with the ethical standards of the Helsinki Declaration. This study was approved by the Institutional Review Board of Yeouido St. Mary’s Hospital, The Catholic University of Korea (No. SC21ZISE0073). Informed consent was waived by IRB of Yeouido St. Mary’s Hospital because anonymous and deidentified information was used for the analysis. Overall, the time between the 2 consecutive biennial health examinations was 2.0 ± 0.5 years.

### Measurements and definitions

Smoking status was determined using a self-reported questionnaire during the health screening examinations at the time of the DM diagnosis (first health examination) and 2 years later (second health examination). The self-reported questionnaire required the participant to choose between being a current smoker, past smoker, or never smoker according to their current smoking status at the time of the examination. Based on the smoking status before and after the DM diagnosis, all participants were grouped into 4 smoking groups as continuous smokers, quitters, new smokers, and nonsmokers. Continuous smokers were those who were current smokers both before and after their DM diagnosis. Quitters were participants who were current smokers before their DM diagnosis but had quit afterwards. New smokers were those who were not current smokers before their DM diagnosis but became current smokers afterwards. Finally, nonsmokers were participants who were not current smokers both before and after their DM diagnosis. Nonsmokers included ex-smokers who had stopped smoking at any time point before their DM diagnosis (first health examination). The amount of smoking was recorded as pack-years by multiplying the average cigarette consumption per day (pack) by the smoking period (years).

The questionnaire section on exercise comprised 3 questions asking about the frequency (days per week) of light, moderate, and vigorous exercise during a recent week: the frequency of light-intensity exercise (e.g., walking slowly or sweeping carpets) for > 30 min, moderate-intensity exercise (e.g., brisk walking, tennis doubles, or bicycling leisurely) for > 30 min, and vigorous-intensity exercise (e.g., running, climbing, fast cycling, or aerobics) for > 20 min. Regular exercise was defined as moderate-intensity exercise for > 30 min at least 5 times per week, and vigorous exercise was defined as vigorous-intensity exercise for > 20 min at least 3 times per week. The study population was categorized into 4 groups according to exercise status at the time of the health examinations before and after the DM diagnosis: nonexercisers, new exercisers, exercise dropouts, and constant exercisers. Nonexercisers were participants who did not exercise both before and after their DM diagnosis. New exercisers were those who did not exercise before but began exercising after their DM diagnosis. Exercise dropouts were participants who exercised before but did not exercise after their DM diagnosis. Constant exercisers were those who continued to exercise both before and after their DM diagnosis.

Figure S1 summarizes our study’s overall configuration. To evaluate whether we could identify an optimal exercise amount related to the best cardiovascular outcomes, we analyzed the hazard ratios (HRs) for the primary outcomes according to stratified energy expenditure. This analysis was performed in the new exerciser group to measure the influence of exercise initiation. To calculate energy expenditure, we rated light-, moderate-, and vigorous-intensity exercise as 2.9, 4.0, and 7.0 metabolic equivalents of task (METs), respectively^13,14^. Exercise-related energy expenditure (MET-min/week) was calculated by summing the product of frequency, intensity, and duration. The total energy expenditure level was stratified into < 500, 500–999, 1000–1499, and ≥ 1500 MET-min/week in an explorative way^13,14^.

### Covariates and primary outcomes

The general medical examination included recording the medical history, completing the self-questionnaire survey, blood pressure measurement, blood sampling, and urinalysis. Blood samples for the measurement of serum glucose and lipid levels were drawn after an overnight fast. Individuals who consumed ≥ 30 g/day of alcohol were defined as heavy alcohol drinkers. Body mass index (BMI) was classified based on the World Health Organization’s Asia–Pacific criteria, with that of 25 kg/m^2^ or greater indicating obesity^15,16^.

The end points of the study were newly diagnosed MI, stroke, or death. MI was defined according to the ICD-10 codes I21 or I22 newly recorded during hospitalization. Stroke was defined according to ICD-10 codes I63 or I64 during hospitalization with claims for brain magnetic resonance imaging or brain computed tomography. The cohort database is linked to the death registration database of Statistics Korea. Participants without MI or stroke during their follow-up were considered to have completed the study at the date of their death or at the end of follow-up, whichever came first. The study population was followed from the baseline to the date of death or cardiovascular events, or until December 31, 2018, whichever came first.

### Statistical analysis

Baseline characteristics are presented as the mean and standard deviation (SD), median (25–75%), or number (%). Participants were classified into 4 groups according to their changes in smoking or exercise status after their diagnosis of DM. The incidence rates of all-cause mortality, MI, and stroke were calculated by dividing the number of incident cases by the total follow-up duration (person-years). The Cox proportional-hazards model was used to estimate HRs and 95% confidence intervals (CIs) for all-cause mortality, MI, and stroke according to the changes in smoking or exercise status after the diagnosis of DM. A multivariable-adjusted proportional-hazards model was applied. Model 1 was adjusted for age, sex, BMI, income status, hypertension, dyslipidemia, fasting blood glucose (FBG) level, kidney function (estimated glomerular filtration rate), use of insulin, and number of oral hypoglycemic agents. Model 2 was adjusted further for alcohol intake and other lifestyle behaviors (smoking or exercise status). The potential modification by age, sex, and obesity was evaluated using stratified analysis and interaction testing using a likelihood ratio test. Statistical analyses were performed using SAS software (version 9.4; SAS Institute, Cary, NC, USA), and a *P* value < 0.05 was considered to be significant.

## Results

### Baseline characteristics of the participants

Overall, 181,591 people were included in the analysis. Their mean age was 57.1 years (Table 1, Table S1); 60.8% were men, 54% had hypertension, and ~ 50% had dyslipidemia. About 17% of patients used ≥ 3 antidiabetic medications. The baseline characteristics according to the change in exercise habits are shown in Table 1 for the population grouped into the 4 exercise categories: nonexercisers (n = 119,895, 66.0%), new exercisers (n = 27,984, 15.4%), exercise dropouts (n = 19,226, 10.6%), and constant exercisers (n = 14,486, 8.0%). The group of nonexercisers were younger, included fewer men, had a higher percentage of smokers, were more likely to be taking insulin or ≥ 3 oral hypoglycemic agents, and had a higher FBG level.

**Table 1:** Characteristics of the study population at the second health examination according to the change in exercise status.

	Total population	Nonexercisers	New exercisers	Exercise dropouts	Constant exercisers
N	181,591	119,895 (66.0%)	27,984 (15.4%)	19,226 (10.6%)	14,486 (8.0%)
Age	57.1 ± 10.9	56.9 ± 11.14	56.62 ± 10.29	58.65 ± 10.33	58.22 ± 9.91
Sex, men	110,488 (60.8)	70,483 (58.8)	17,950 (64.1)	11,883 (61.8)	10,172 (70.2)
Low-income level	28,866 (15.9)	60,048 (50.1)	13,517 (48.3)	9382 (48.8)	6883 (47.5)
Hypertension	98,652 (54.3)	65,214 (54.39)	14,601 (52.18)	10,843 (56.4)	7994 (55.18)
Dyslipidemia	89,830 (49.5)	60,048 (50.1)	13,517 (48.3)	9382 (48.8)	6883 (47.51)
**Pharmacologic therapy for DM**					
Insulin	19,772 (10.89)	13,301 (11.1)	3045 (10.9)	2046 (10.6)	1380 (9.5)
Number of OHA					
≤ 1	75,352 (41.5)	48,760 (40.6)	11,614 (41.5)	8340 (43.4)	6638 (45.8)
2	75,217 (41.4)	49,923 (41.6)	11,713 (41.9)	7774 (40.4)	5807 (40.1)
≥ 3	31,022 (17.1)	21,212 (17.7)	4657 (16.6)	3112 (16.2)	2041 (14.1)
Medication					
Metformin	162,029 (89.2)	107,071 (89.3)	25,029 (89.4)	16,997 (88.4)	12,932 (89.3)
Sulfonylurea	85,205 (46.9)	57,351 (47.8)	12,963 (46.3)	8830 (45.9)	6061 (41.8)
DPPIV-inhibitors	49,110 (27.0)	32,738 (27.3)	7635 (27.3)	5029 (26.2)	3708 (25.6)
TZD	10,562 (5.8)	7126 (5.9)	1650 (5.9)	1051 (5.5)	735 (5.1)
AGI	10,741 (5.9)	7275 (6.1)	1595 (5.7)	1121 (5.8)	750 (5.2)
Body mass index (kg/m^2^)	25.3 ± 3.35	25.4 ± 3.4	25.1 ± 3.2	25.2 ± 3.3	25.0 ± 3.0
Systolic BP (mmHg)	127.1 ± 14.5	127.1 ± 14.6	126.6 ± 14.4	127.5 ± 14.6	127.5 ± 14.0
Diastolic BP (mmHg)	78.5 ± 9.6	78.6 ± 9.7	78.1 ± 9.5	78.4 ± 9.6	78.4 ± 9.3
Total cholesterol (mg/dL)	190.2 ± 42.4	191.1 ± 42.6	187.7 ± 40.2	189.7 ± 44.6	187.9 ± 42.2
Fasting blood glucose (mg/dL)	132.2 ± 40.6	133.3 ± 42.0	128.5 ± 36.6	132.1 ± 40.3	129.8 ± 35.5
eGFR (mL/min/1.73 m^2^)	89.8 ± 39.5	89.9 ± 38.6	89.8 ± 41.3	88.9 ± 36.8	89.2 ± 46.3
Current smoking	44,366 (24.43)	31,213 (26.03)	6180 (22.08)	4132 (21.49)	2841 (19.61)
Heavy alcohol consumption	17,001 (9.36)	11,358 (9.47)	2422 (8.65)	1693 (8.81)	1528 (10.55)
Use of statin	85,935 (47.3)	57,024 (47.6)	13,248 (47.3)	8963 (46.6)	6700 (46.3)
Use of anti-hypertensive drugs	95,884 (52.8)	63,035 (52.6)	14,330 (51.2)	10,716 (55.7)	7803 (53.9)
Use of aspirin	47,909 (26.4)	31,426 (26.2)	7222 (25.8)	5317 (27.7)	3944 (27.2)

Data are expressed as the means ± SD, or n (%).

*DM* diabetes mellitus, *OHA* oral hypoglycemic agents, *DPPIV-inhibitors* dipeptidyl peptidase IV-inhibitors, *TZD* thiazolidinedione, *AGI* alpha glucosidase inhibitors, *BP* blood pressure, *eGFR* estimated glomerular filtration rate.

The baseline characteristics according to the change in smoking habits are shown in Table S1. The study population was classified into 4 categories according to smoking habits: nonsmokers (n = 126,854, 69.9%), new smokers (n = 6,379, 3.5%), quitters (n = 10,371, 5.7%), and continuous smokers (n = 37,987, 20.9%). The group of current smokers were younger, included more men, were more likely to be taking ≥ 3 oral hypoglycemic agents, had a higher FBG level, and had a lower percentage of regular exercisers.

### Effects of changes in exercise habits on the risk of CVD and mortality

The median follow-up was 6.07 (5.09–7.02) years. Initiating exercise after the DM diagnosis was associated with a lower risk of MI, stroke, and all-cause mortality compared with the nonexerciser group: the HR (95% CI) was 0.85 (0.76–0.94) for MI, 0.86 (0.78–0.94) for stroke, and 0.84 (0.89–0.90) for all-cause mortality (Table 2). CVD mortality was reduced by 20% by engaging in moderate or vigorous physical activity after the DM diagnosis: the HR (95% CI) was 0.80 (0.68–0.94) for CVD mortality.

**Table 2:** Hazard ratios with 95% confidence intervals for myocardial infarction, stroke, and all-cause or cardiovascular (CV) mortality according to the change in exercise status.

Outcome and exercise group	Number of individuals (n)	Number of events (n)	Incidence rate (per 1000 person-years)	Model 1	Model 2
**Myocardial infarction**					
Nonexercisers	119,895	2148	3.00	1 (ref.)	1 (ref.)
New exercisers	27,984	410	2.43	0.82 (0.74, 0.91)	**0.85 (0.76, 0.94)**
Exercise dropouts	19,226	349	3.03	0.93 (0.83, 1.04)	0.95 (0.85, 1.06)
Constant exercisers	14,486	191	2.19	0.68 (0.58, 0.79)	0.72 (0.62, 0.83)
**Stroke**					
Nonexercisers	119,895	2902	4.07	1 (ref.)	1 (ref.)
New exercisers	27,984	545	3.24	0.83 (0.76, 0.91)	**0.86 (0.78, 0.94)**
Exercise dropouts	19,226	428	3.73	0.82 (0.74, 0.91)	0.84 (0.76, 0.93)
Constant exercisers	14,486	256	2.95	0.67 (0.59, 0.76)	0.70 (0.62, 0.80)
**All-cause mortality**					
Nonexercisers	119,895	5255	7.29	1 (ref.)	1 (ref.)
New exercisers	27,984	1005	5.93	0.82 (0.77, 0.88)	**0.84 (0.79, 0.90)**
Exercise dropouts	19,226	874	7.53	0.88 (0.82, 0.95)	0.90 (0.84, 0.97)
Constant exercisers	14,486	544	6.22	0.73 (0.67, 0.80)	0.76 (0.70, 0.84)
**CV mortality**					
Nonexercisers	119,895	1001	1.39	1 (ref.)	1 (ref.)
New exercisers	27,984	173	1.02	0.78 (0.66, 0.91)	**0.80 (0.68, 0.94)**
Exercise dropouts	19,226	160	1.38	0.86 (0.73, 1.02)	0.88 (0.74, 1.04)
Constant exercisers	14,486	89	1.02	0.66 (0.53, 0.82)	0.69 (0.55, 0.86)

Model 1; adjusted for age, sex, body mass index, income status, hypertension, dyslipidemia, fasting blood glucose, kidney function, use of insulin, and number of oral hypoglycemic agents.

Model 2; model 1 + adjusted for alcohol intake and smoking status.

The exercise dropout group exhibited a slightly lower risk of stroke and all-cause mortality compared with the nonexerciser group, the HR (95% CI) was 0.84 (0.76–0.93) for stroke and 0.90 (0.84–0.97) for all-cause mortality. The exercise dropout group exhibited similar risks for MI and CVD mortality as the nonexerciser group. The constant exerciser group had a lower risk of all-cause mortality and CVD mortality compared with the nonexerciser group. The HR (95% CI) was 0.76 (0.70–0.84) for all-cause mortality and 0.69 (0.55–0.86) for CVD mortality. Further adjustments to the use of statin, anti-hypertensive drugs and aspirin for the model did not alter the associations (data not shown).

**Figure 1:**
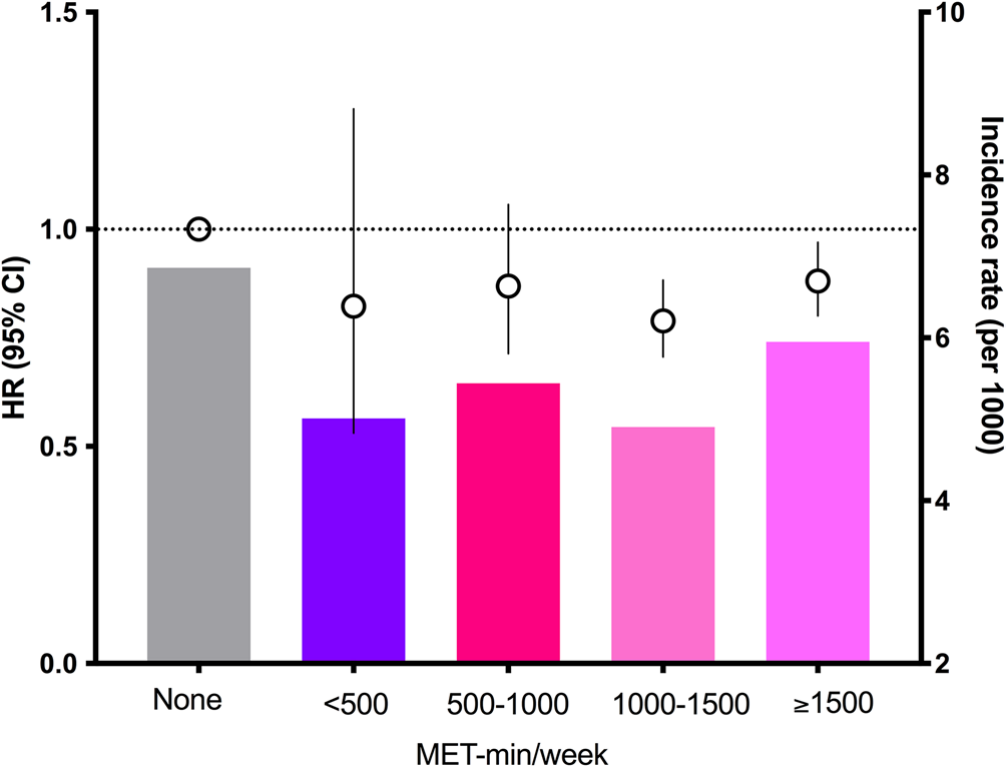
Incidence rates, hazard ratios, and 95% confidence intervals for cardiovascular disease in the new exerciser group according to energy expenditure (MET-min/week). The reference group was defined as the nonexerciser group. The analysis was adjusted for age, sex, body mass index, income status, hypertension, dyslipidemia, fasting blood glucose level, kidney function, use of insulin, number of oral hypoglycemic agents, alcohol intake, and smoking status.

To evaluate the exercise amount associated with a reduced risk of CVD, we analyzed the HR of CVD according to stratified energy expenditure. The reference group was defined as the nonexerciser group. CVD risk was lowest in the group with an exercise amount of 1000–1500 MET-min/week (HR 0.79; 95% CI 0.71–0.88; Fig. 1). CVD risk was also lower in the group with higher amounts of exercise (> 1500 MET-min/week) compared with the nonexerciser group (HR 0.88; 95% CI 0.80–0.97).

### Effects of changes in smoking habits on the risk of CVD and mortality

Continuous smokers had an 82% higher risk of MI and 71% higher risk of stroke compared with nonsmokers. The HR (95% CI) was 1.82 (1.65–2.00) for MI and 1.71 (1.57–1.86) for stroke (Table 3). The risk of all-cause mortality was 80% higher in continuous smokers than in nonsmokers: HR 1.80 (95% CI 1.69–1.91) (*P* < 0.001). People who had quit smoking after the DM diagnosis (quitters) had a higher risk of CVD and all-cause mortality compared with the nonsmokers. The HR (95% CI) was 1.42 (1.22–1.65) for MI, 1.32 (1.16–1.51) for stroke, and 1.68 (1.55–1.84) for all-cause mortality. Although quitters still had a higher risk of MI, stroke, and all-cause mortality than nonsmokers, their risk was much lower than that in continuous smokers (Table 3).

**Table 3:** Hazard ratios with 95% confidence intervals for myocardial infarction, stroke, and all-cause or cardiovascular (CV) mortality according to the change in smoking status.

Outcome and smoking group	Number of individuals (n)	Number of events (n)	Incidence rate (per 1000 person-years)	Model 1	Model 2
**Myocardial infarction**					
Nonsmokers	126,854	1971	2.59	1 (ref.)	1 (ref.)
New smokers	6379	139	3.63	1.70 (1.43, 2.03)	1.76 (1.47, 2.10)
Quitters	10,371	203	3.27	1.42 (1.23, 1.65)	1.42 (1.22, 1.65)
Continuous smokers	37,987	785	3.47	1.77 (1.61, 1.95)	1.82 (1.65, 2.00)
**Stroke**					
Nonsmokers	126,854	2772	3.66	1 (ref.)	1 (ref.)
New smokers	6379	162	4.23	1.54 (1.31, 1.82)	1.53 (1.30, 1.79)
Quitters	10,371	246	3.98	1.33 (1.16, 1.52)	1.32 (1.16, 1.51)
Continuous smokers	37,987	951	4.22	1.74 (1.60, 1.90)	1.71 (1.57, 1.86)
**All-cause mortality**					
Nonsmokers	126,854	4808	6.29	1 (ref.)	1 (ref.)
New smokers	6379	308	7.97	1.48 (1.31, 1.66)	1.48 (1.32, 1.67)
Quitters	10,371	640	10.24	1.70 (1.56, 1.85)	1.68 (1.55, 1.84)
Continuous smokers	37,987	1922	8.43	1.80 (1.72, 1.91)	1.80 (1.69, 1.91)
**CV mortality**					
Nonsmokers	126,854	968	1.27	1 (ref.)	1 (ref.)
New smokers	6379	50	1.29	1.40 (1.05, 1.87)	1.41 (1.05, 1.88)
Quitters	10,371	101	1.62	1.58 (1.28, 1.95)	1.57 (1.27, 1.94)
Continuous smokers	37,987	304	1.33	1.78 (1.54, 2.05)	1.77 (1.53, 2.04)

Model 1; adjusted for age, sex, body mass index, income status, hypertension, dyslipidemia, fasting blood glucose, kidney function, use of insulin, and number of oral hypoglycemic agents.

Model 2; model 1 + adjusted for alcohol intake and exercise status.

A history of smoking < 10 pack-years among quitters was not associated with a higher risk of CVD compared with nonsmokers (Table 4). A history of smoking > 10 pack-years among quitters after the diagnosis of DM was associated with a higher risk of CVD compared with nonsmokers. The HRs (95% CI) for quitters with a smoking history of 10–20, 20–30, and ≥ 30 pack-years were 1.33 (1.08–1.63), 1.39 (1.14–1.70), and 1.36 (1.15–1.60), respectively. However, these HRs were lower than those for continuous smokers regardless of the amount of smoking. The HRs (95% CI) for continuous smokers with a smoking history of 10–20, 20–30, and > 30 pack-years were 1.66 (1.48–1.87), 1.86 (1.68–2.07), and 1.82 (1.67–1.98). Among new smokers, there was a dose-dependent association between smoking amount and CVD and all-cause mortality risks.

**Table 4:** Hazard ratios for myocardial infarction, stroke, and all-cause mortality in each group stratified by the smoking amount.

Smoking group	Pack-years	Number of individuals (n)	Number of events (n)	Incidence rate (per 1000 person-years)	Model 2
**MI or stroke**					
Nonsmokers	0	126,854	4567	6.07	1 (ref.)
New smokers	< 10	1874	69	6.15	1.53 (1.21, 1.95)
	10–20	1719	69	6.75	1.55 (1.22, 1.97)
	20–30	1216	55	7.63	1.62 (1.24, 2.11)
	≥ 30	1570	95	10.29	1.74 (1.42, 2.14)
Quitters	< 10	2620	81	5.18	1.22 (0.98, 1.52)
	10–20	2601	94	6.07	1.33 (1.08, 1.63)
	20–30	2351	100	7.11	1.39 (1.14, 1.70)
	≥ 30	2799	147	9.08	1.36 (1.15, 1.60)
Continuous smokers	< 10	5668	180	5.35	1.59 (1.37, 1.85)
	10–20	10,038	348	5.83	1.66 (1.48, 1.87)
	20–30	9664	418	7.32	1.86 (1.68, 2.07)
	≥ 30	12,617	734	10.03	1.82 (1.67, 1.98)
**All-cause mortality**					
Nonsmokers	0	126,854	4808	6.29	1 (ref.)
New smokers	< 10	1874	61	5.36	1.27 (0.99, 1.64)
	10–20	1719	74	7.11	1.43 (1.14, 1.80)
	20–30	1216	56	7.60	1.35 (1.03, 1.75)
	≥ 30	1570	117	12.35	1.77 (1.47, 2.13)
Quitters	< 10	2620	101	6.37	1.47 (1.21, 1.79)
	10–20	2601	131	8.31	1.60 (1.34, 1.90)
	20–30	2351	158	11.04	1.77 (1.50, 2.07)
	≥ 30	2799	250	15.06	1.79 (1.58, 2.04)
Continuous smokers	< 10	5668	218	6.39	1.94 (1.69, 2.22)
	10–20	10,038	396	6.54	1.83 (1.65, 2.04)
	20–30	9664	434	7.46	1.76 (1.59, 1.94)
	≥ 30	12,617	874	11.65	1.77 (1.64, 1.92)

The smoking amount of new and continuous smokers was calculated based on the questionnaire from the second health examination. The smoking amount of quitters was calculated based on the questionnaire at the first health examination because they had quit smoking before the second health examination.

Adjusted for age, sex, body mass index, income status, hypertension, dyslipidemia, fasting blood glucose, kidney function, use of insulin, number of oral hypoglycemic agents, alcohol drinking and exercise status.

### Combined effects of changes in smoking and exercise habits on the risk of CVD

**Figure 2:**
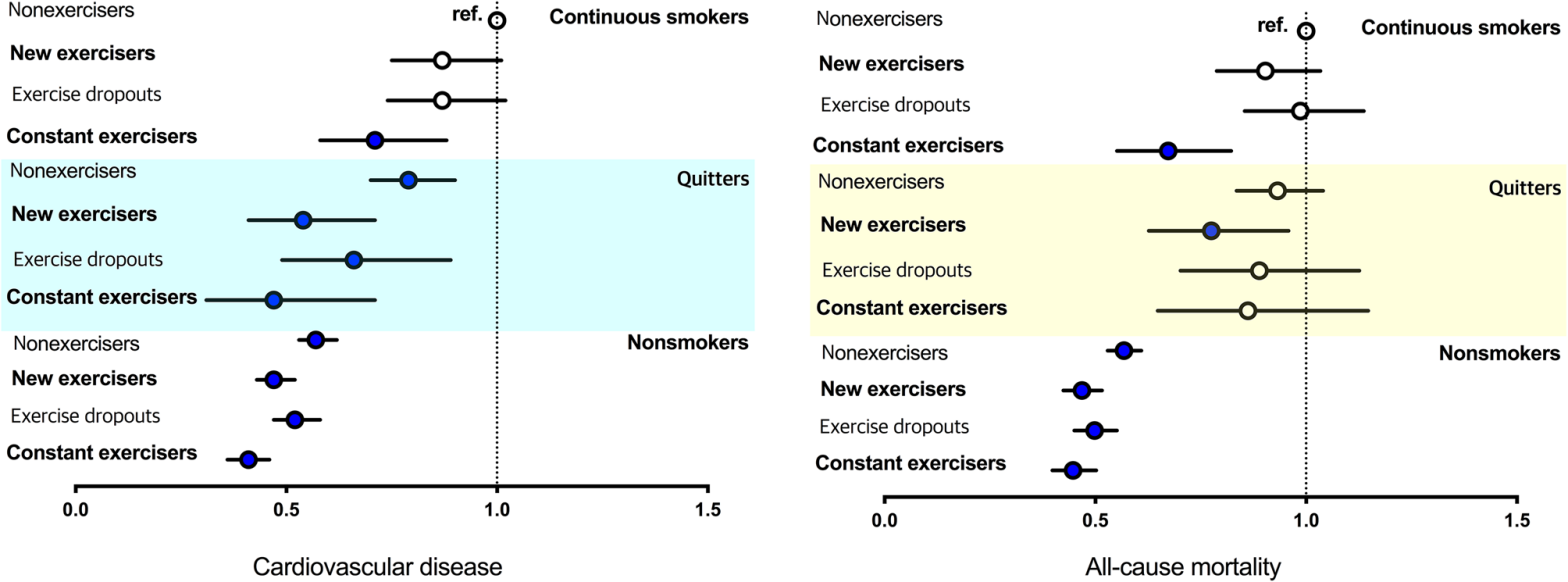
Hazard ratios and 95% confidence intervals for cardiovascular disease (left panel) and all-cause mortality (right panel) according to the changes in smoking and exercise habits after the diagnosis of diabetes mellitus. The group of continuous smokers and nonexercisers was defined as the reference group. The analysis was adjusted for age, sex, body mass index, income status, hypertension, dyslipidemia, fasting blood glucose level, kidney function, use of insulin, number of oral hypoglycemic agents, and alcohol intake.

The group of continuous smokers and nonexercisers was considered as the reference group. People who currently smoked and had initiated exercise had no significant reduction in the risk of CVD (HR 0.87; 95% CI 0.75–1.01) and all-cause mortality (HR 0.90; 95% CI 0.79–1.03) (Fig. 2). Those who quit smoking and remained nonexercisers had a 21% reduced risk of CVD (HR 0.79; 95% CI 0.70–0.90). People who quit smoking and started exercising had a 46% reduction in the risk of CVD (HR 0.54; 95% CI 0.41–0.71) and a 22% reduction in the risk of all-cause mortality (HR 0.78; 95% CI 0.63–0.96). For those who did not smoke and continued to exercise (nonsmokers and constant exercisers), the risk of CVD was reduced by 59% (HR 0.41; 95% CI 0.36–0.46) and all-cause mortality was reduced by 55% (HR 0.45; 95% CI 0.40–0.50).

### Subgroup and sensitivity analysis

The decrease in the risk of CVD was larger in the constant and new exerciser groups among those aged ≥ 65 years compared with those aged < 65 years (*P* for interaction = 0.038). The decrease in the risk of CVD was larger in the constant or new exerciser among those without obesity (*P* for interaction = 0.017; Table S2). The HRs for CVD and all-cause mortality were larger in the younger people in the continuous smoker group (*P* for interaction = 0.001; Table S3). The highest HR for CVD was observed in the continuous smokers aged < 65 years (HR 1.94, 95% CI 1.79–2.11). The interaction between the risk of all-cause mortality and age may be explained by the greater benefits of smoking cessation in younger patients.

Sensitivity analysis was performed to exclude people whose end point occurred within 1 year of the follow-up to account for the possibility of reverse causation. However, the results were similar after excluding these people (Table S4). To account for the possible influence of survival, we performed another sensitivity analysis by excluding participants with a history of malignancy. The associations between the changes in exercise or smoking habit and all-cause mortality were similar after excluding these people (Table S5).

## Discussion

The main finding of this study is that smoking cessation and starting exercise after the diagnosis of type 2 DM were associated with a reduced risk of CVD and all-cause mortality compared with continuous smoking and not exercising. In this population, people who engaged in exercise only after their diagnosis of DM had a 15% lower risk of CVD and a 16% lower risk of all-cause mortality than those who did not. Patients who quit smoking and started exercising had a 46% reduction in the risk of CVD and a 22% lower risk of all-cause mortality than those who did not.

Even though exercise is considered a cornerstone in the treatment of diabetes, few studies have investigated its relationship with CVD risk and mortality in people with diabetes^9^. Exercise intervention studies show that physical activity can improve insulin sensitivity, glycemic control, and lipoprotein profile among people with type 2 DM^9,17^. Most metabolic studies have reported significant effects of exercise on glucose control and triglyceride level. However, in the Look AHEAD study, which investigated the effects of an intensive lifestyle intervention on CVD in overweight people with type 2 DM, the interventions did not reduce incidence of CVD, although they had a positive effect on cardiovascular risk factors^17^. The evidence of an association between exercise and a reduction in mortality or CVD is mixed^9^. An association between walking and mortality in adults with diabetes is inconsistent, and only moderate-intensity walking appears to be associated with a reduced risk of all-cause and CVD mortality^18^.

The mortality rates have been reported to be lowest for people who walk 3–4 h/week and those whose walking involves moderate increases in heart and breathing rates^18^. In an observational cohort study, cycling was associated with lower all-cause and CVD mortality risk among people with DM independent of participation in other types of physical activity^19^. In our study, a level of 1000 MET-min/week was identified as the minimum requirement for initiating exercise in patients with newly diagnosed DM. Current guidelines recommend 500–1000 MET-min/week of regular physical activity^20^. The current Public Health Guidelines for Physical Activity recommend that adults accumulate a minimum of 150 min/week of moderate-intensity physical activity or a minimum of 75 min/week of vigorous-intensity physical activity^20^. A meta-analysis has reported that 250 MET-min/week, which corresponds to 75 min/week or 15 min/day on 5 days, has a beneficial effect on health in older adults^21^. In our study, people who exercised 500–1000 MET-min/week were not observed to have lower risks of CVD and all-cause mortality than those who did not exercise. The frequency of exercise associated with the lowest risk of all-cause mortality is 5–6 times per week for people with DM versus 3–4 times per week in those without DM^22^. The differences in optimal exercise amounts appear to be related to differences in the populations studied; for example, in our study, the participants were middle-aged people with newly diagnosed type 2 DM.

In our study, 24% of people with newly diagnosed DM remained current smokers; this percentage is similar to that reported in US representative samples^23^. Smoking cessation remains a major treatment target for people with diabetes despite the concern about smoking cessation in people with diabetes because of the resultant weight gain and deterioration in glycemic control^6,24^. Quitting smoking may increase the risk of new-onset type 2 DM, even if this risk tends to decrease progressively over the long term^6^. Smoking cessation contributes to an increased risk of new-onset type 2 DM in the first and second years after quitting smoking (relative risk = 1.83 and 2.02, respectively), although the risk declines rapidly thereafter^24^. In our study, cessation of smoking after the diagnosis of type 2 DM had beneficial effects on the risks of CVD and mortality.

A meta-analysis reported that the risk of macrovascular complications is higher for people with diabetes who smoke than for those who do not^5^. In the subgroup analysis in our study, a higher HR for all-cause mortality was observed in women who quit smoking compared with those who currently smoked (Table 4). The “sick-quitter” phenomenon may contribute to this association between smoking cessation and mortality, but this remains controversial. To lessen the chance of reverse causality, we repeated the analysis after excluding participants with a history of cancer at the baseline and after excluding those whose end points occurred within 1 year. The results of these analyses showed that the HR for all-cause mortality was lower in people who quit smoking than in those who continued to smoke. Despite the possible bias because of the sick-quitter phenomenon, we have shown that smoking cessation can reduce the risk of mortality in patients with newly diagnosed type 2 DM. However, after smoking cessation, the risk of CVD did not decrease to the same level as that in nonsmokers.

The HRs for CVD or all-cause mortality in continuous smokers were larger in the younger age groups. We observed a significant interaction between the risk of all-cause mortality and age, which might be explained by the greater benefits of smoking cessation in younger people. We also observed no reduction in the risks of CVD and mortality in people who started exercise but continued to smoke, which suggests that both smoking cessation and initiation of exercise are needed to reduce CVD and mortality after the diagnosis of type 2 DM. Recent research has suggested that smoking cessation, but not reduction, is associated with reduced CVD risk^25^.

The current study has several strengths. The most important is that these data were based on a nationwide population study covering nearly 100% of all Koreans with type 2 DM. This was a large-scale epidemiologic study that included 180,000 people to investigate an issue that cannot be examined in RCTs. Second, the study included people who were newly diagnosed with type 2 DM. Lifestyle modification during the initial period after the diagnosis of type 2 DM is critical for improving glycemic control and risk factors. In the initial period after the DM diagnosis, people may be more amenable to adopting a healthy lifestyle.

This study also has limitations. First, this was an observational study and therefore, the associations between changes in smoking and exercise habits and end points may not be causal. Second, participants who continued to smoke and remained inactive even after the DM diagnosis may have had less interest in improving their health, which may have led to poor diabetes control or other unhealthy behaviors, and may explain their increased risk. Third, we cannot discount the possible effects of unmeasured confounding variables. Finally, we could not adjust for changes in smoking and exercise habits during follow-up, because we could not obtain time-varying confounders in our dataset. These changes in study participants during follow-up period should be considered in future studies.

In conclusion, initiation and maintenance of exercise after a diagnosis of DM were associated with a significantly lower risk of MI, stroke, and all-cause mortality. Smoking cessation and maintenance of nonsmoking after the DM diagnosis were also associated with significantly lower risk of MI, stroke, and all-cause mortality. Smoking cessation and initiation of exercise after the DM diagnosis was associated with a 46% reduced risk of CVD.

## Supplementary Information

Supplementary Information.

## Data Availability

The datasets used and/or analyzed in the current study are available from the corresponding authors (MKK and HSK) upon reasonable request.
