# Supplementary material for: Effects of exercise initiation and smoking cessation after new-onset type 2 diabetes mellitus on risk of mortality and cardiovascular outcomes
Source: Sci Rep. 2022 Jun 23;12:10656. doi: 10.1038/s41598-022-14603-1 (PMC9226070; doi:10.1038/s41598-022-14603-1)
Supplement: Supplementary file 1 — Supplementary Information. [file 41598_2022_14603_MOESM1_ESM.docx]

**Table S1.** Characteristics of the study population at the second health examination according to the change in smoking status.

|  | Total population | Nonsmokers | New smokers | Quitters | Continuous smokers |
| --- | --- | --- | --- | --- | --- |
| N | 181591 | 126854 (69.9%) | 6379 (3.5%) | 10371 (5.7%) | **37987 (20.9%)** |
| Age | 57.1 ± 10.9 | 59.0 ± 10.6 | 53.9 ± 10.4 | 54.6 ± 10.5 | 52.2 ± 10.1 |
| Sex, men | 110488 (60.8) | 59010 (46.5) | 5792 (90.8) | 9410 (90.7) | 36276 (95.5) |
| Low-income level | 28866 (15.9) | 20678 (16.3) | 922 (14.5) | 1504 (14.5) | 5762 (15.2) |
| Hypertension | 98652 (54.3) | 71644 (56.5) | 3215 (50.4) | 5289 (51.0) | 18504 (48.7) |
| Dyslipidemia | 89830 (49.5) | 63794 (50.3) | 2970 (46.6) | 5086 (49.0) | 17980 (47.3) |
| Pharmacologic therapy for DM | |  |  |  |  |
| Insulin | 19772 (10.89) | 13508 (10.7) | 657 (10.3) | **1710 (16.5)** | 3897 (10.3) |
| Number of OHA |  |  |  |  |  |
| ≤1 | 75352 (41.5) | 55443 (43.7) | 2403 (37.7) | 3838 (37.0) | 13668 (35.3) |
| 2 | 75217 (41.4) | 50964 (40.2) | 2762 (43.3) | 4453 (42.9) | **17038 (44.9)** |
| ≥3 | 31022 (17.1) | 20447 (16.1) | 1214 (19.0) | 2080 (20.1) | **7281 (19.8)** |
| Medication |  |  |  |  |  |
| Metformin | 162029 (89.2) | 112803 (88.9) | 5773 (90.5) | 9163 (88.4) | 34290 (90.3) |
| Sulfonylurea | 85205 (46.9) | 57351 (45.2) | 3177 (49.8) | 5242 (50.5) | 19435 (51.2) |
| DPPIV-inhibitors | 49110 (27.0) | 32982 (26.0) | 1809 (28.4) | 3012 (29.0) | 11307 (29.8) |
| TZD | 10562 (5.8) | 7068 (5.6) | 426 (6.7) | 647 (6.2) | 2421 (6.4) |
| AGI | 10741 (5.9) | 7385 (5.8) | 407 (6.4) | 710 (6.9) | 2239 (5.9) |
| Body mass index | 25.3 ± 3.35 | 25.3 ± 3.4 | 25.3 ± 3.2 | 25.3 ± 3.2 | 25.2 ± 3.4 |
| Systolic BP | 127.1 ± 14.5 | 127.4 ± 14.7 | 126.0 ± 14.2 | 126.5 ± 14.0 | 126.3 ± 14.1 |
| Diastolic BP | 78.5 ± 9.6 | 78.3 ± 9.6 | 78.6 ± 9.8 | 78.7 ± 9.6 | 79.1 ± 9.7 |
| Total cholesterol (mg/dL) | 190.2 ± 42.4 | 189.9 ± 42.4 | 189.8 ± 41.8 | 189.7 ± 42.8 | 191.3 ± 42.6 |
| Fasting blood glucose (mg/dl) | 132.2 ± 40.6 | 128.8 ± 36.7 | 136.3 ± 44.5 | 136.9 ± 45.0 | **141.4 ± 48.8** |
| eGFR | 89.8 ± 39.5 | 88.6 ± 38.7 | 91.3 ± 36.0 | 90.5 ± 40.0 | 93.3 ± 42.4 |
| Regular exercise | 44366 (24.4) | 30835 (24.3) | 1418 (22.2) | 2614 (25.2) | 7603 (20.01) |
| Heavy Alcohol consumption | 17001 (9.36) | 7279 (5.74) | 1091 (17.1) | 1250 (12.05) | 7381 (19.43) |

Data are expressed as the means ± SD, or n (%). All variables were significantly different among the groups (all P<0.001).

DM, diabetes mellitus; OHA, oral hypoglycemic agents; DPPIV-inhibitors, dipeptidyl peptidase IV-inhibitors;TZD, thiazolidinedione; AGI, alpha glucosidase inhibitors; BP, blood pressure; eGFR, estimated glomerular filtration rate

**Table S2**. Subgroup analyses of association between changes in exercise habits and cardiovascular disease (CVD) and all-cause mortality

|  | Outcome and exercise group | Number of individuals (n) | Number of events (n) | Incidence rate  (per 1000 person-years) | HR (95% CI)* | P for interaction |
| --- | --- | --- | --- | --- | --- | --- |
| **CVD** (myocardial infarction or stroke) | | |  |  |  |  |
| < 65 years | Nonexercisers | 90191 | 2460 | 4.56 | 1 (ref.) | 0.038 |
|  | New exercisers | 21734 | 519 | 3.96 | 0.90 (0.82, 0.99) |  |
|  | Exercise dropouts | 13642 | 391 | 4.76 | 1.01 (0.90, 1.12) |  |
|  | Constant exercisers | 10559 | 220 | 3.45 | 0.75 (0.65, 0.86) |  |
| ≥65 years | Nonexercisers | 29704 | 2399 | 14.25 | 1 (ref.) |  |
|  | New exercisers | 6250 | 392 | 10.86 | 0.80 (0.72, 0.89) |  |
|  | Exercise dropouts | 5584 | 367 | 11.50 | 0.82 (0.73, 0.91) |  |
|  | Constant exercisers | 3927 | 209 | 9.23 | 0.68 (0.59, 0.78) |  |
| Men | Nonexercisers | 70483 | 2949 | 7.10 | 1 (ref.) | 0.245 |
|  | New exercisers | 17950 | 647 | 6.06 | 0.87 (0.80, 0.94) |  |
|  | Exercise dropouts | 11883 | 529 | 7.55 | 0.94 (0.86, 1.03) |  |
|  | Constant exercisers | 10172 | 338 | 5.57 | 0.73 (0.65, 0.82) |  |
| Women | Nonexercisers | 49412 | 1910 | 6.52 | 1 (ref.) |  |
|  | New exercisers | 10034 | 264 | 4.38 | 0.80 (0.71, 0.91) |  |
|  | Exercise dropouts | 7343 | 229 | 5.21 | 0.83 (0.72, 0.95) |  |
|  | Constant exercisers | 4314 | 91 | 3.54 | 0.64 (0.52, 0.79) |  |
| BMI<25 kg/m^2^ | Nonexercisers | 57297 | 2547 | 7.59 | 1 (ref.) | 0.017 |
|  | New exercisers | 14219 | 456 | 5.39 | 0.77 (0.70, 0.85) |  |
|  | Exercise dropouts | 9699 | 390 | 6.81 | 0.84 (0.76, 0.94) |  |
|  | Constant exercisers | 7568 | 220 | 4.90 | **0.64 (0.56, 0.74)** |  |
| BMI ≥ 25 kg/m^2^ | Nonexercisers | 62598 | 2312 | 6.21 | 1 (ref.) |  |
|  | New exercisers | 13765 | 455 | 5.52 | 0.93 (0.84, 1.03) |  |
|  | Exercise dropouts | 9527 | 368 | 6.49 | 0.96 (0.86, 1.07) |  |
|  | Constant exercisers | 6918 | 209 | 5.03 | 0.78 (0.68, 0.90) |  |
| **All-cause Mortality** | | |  |  |  |  |
| < 65 years | Nonexercisers | 90191 | 2049 | 3.75 | 1 (ref.) | 0.038 |
|  | New exercisers | 21734 | 463 | 3.50 | 0.93 (0.84, 1.03) |  |
|  | Exercise dropouts | 13642 | 344 | 4.14 | 1.03 (0.92, 1.15) |  |
|  | Constant exercisers | 10559 | 216 | 3.36 | 0.83 (0.72, 0.95) |  |
| ≥ 65 years | Nonexercisers | 29704 | 3206 | 18.41 | 1 (ref.) |  |
|  | New exercisers | 6250 | 542 | 14.64 | 0.80 (0.73, 0.88) |  |
|  | Exercise dropouts | 5584 | 530 | 16.12 | 0.85 (0.78, 0.93) |  |
|  | Constant exercisers | 3927 | 328 | 14.18 | **0.75 (0.67, 0.84)** |  |
| Men | Nonexercisers | 70483 | 3680 | 8.71 | 1 (ref.) | 0.117 |
|  | New exercisers | 17950 | 819 | 7.55 | 0.88 (0.81, 0.95) |  |
|  | Exercise dropouts | 11883 | 692 | 9.68 | 0.94 (0.86, 1.01) |  |
|  | Constant exercisers | 10172 | 461 | 7.49 | 0.77 (0.70, 0.85) |  |
| Women | Nonexercisers | 49412 | 1575 | 5.29 | 1 (ref.) |  |
|  | New exercisers | 10034 | 186 | 3.05 | 0.75 (0.65, 0.88) |  |
|  | Exercise dropouts | 7343 | 182 | 4.08 | 0.83 (0.71, 0.97) |  |
|  | Constant exercisers | 4314 | 83 | 3.20 | 0.81 (0.65, 1.01) |  |
| BMI<25 kg/m^2^ | Nonexercisers | 57297 | 3390 | 9.92 | 1 (ref.) | 0.239 |
|  | New exercisers | 14219 | 642 | 7.49 | 0.83 (0.76, 0.90) |  |
|  | Exercise dropouts | 9699 | 547 | 9.38 | 0.86 (0.79, 0.95) |  |
|  | Constant exercisers | 7568 | 345 | 7.59 | 0.74 (0.66, 0.82) |  |
| BMI ≥25 kg/m^2^ | Nonexercisers | 62598 | 1865 | 4.92 | 1 (ref.) |  |
|  | New exercisers | 13765 | 363 | 4.34 | 0.90 (0.80, 1.01) |  |
|  | Exercise dropouts | 9527 | 327 | 5.66 | 1.00 (0.82, 1.13) |  |
|  | Constant exercisers | 6918 | 199 | 4.74 | 0.86 (0.74, 0.99) |  |

*Adjusted for age, sex, body mass index, income status, hypertension, dyslipidemia, fasting blood glucose, kidney function, use of insulin, number of oral hypoglycemic agents, alcohol intake and smoking status

**Table S3**. Subgroup analyses of association between changes in smoking habits and cardiovascular disease and all-cause mortality

|  | Outcome and exercise group | Number of individuals (n) | Number of events (n) | Incidence rate  (per 1000 person-years) | HR (95% CI)* | P for interaction |
| --- | --- | --- | --- | --- | --- | --- |
| **CVD** (myocardial infarction or stroke) | | |  |  |  |  |
| < 65 years | Nonsmokers | 88512 | 1873 | 3.52 | 1 (ref.) | <0.001 |
|  | New smokers | 5386 | 199 | 6.15 | 1.86 (1.60, 2.16) |  |
|  | Quitters | 8593 | 263 | 5.09 | 1.41 (1.23, 1.61) |  |
|  | Continuous smokers | 33635 | 1255 | 6.27 | **1.94 (1.79, 2.11)** |  |
| ≥ 65 years | Non-smokers | 38342 | 2694 | 12.24 | 1 (ref.) |  |
|  | New smokers | 993 | 89 | 16.17 | 1.28 (1.03, 1.59) |  |
|  | Quitters | 1778 | 159 | 16.39 | 1.25 (1.06, 1.47) |  |
|  | Continuous smokers | 4352 | 425 | 17.97 | 1.45 (1.30, 1.62) |  |
| Men | Non-smokers | 59010 | 2226 | 6.38 | 1 (ref.) | 0.053 |
|  | New smokers | 5792 | 264 | 7.66 | 1.60 (1.41, 1.83) |  |
|  | Quitters | 9410 | 371 | 6.65 | 1.25 (1.12, 1.40) |  |
|  | Continuous smokers | 36276 | 1602 | 7.50 | 1.72 (1.60, 1.84) |  |
| Women | Non-smokers | 67844 | 2341 | 5.80 | 1 (ref.) |  |
|  | New smokers | 587 | 24 | 7.04 | 1.42 (0.95, 2.12) |  |
|  | Quitters | 961 | 51 | 9.14 | 1.92 (1.46, 2.54) |  |
|  | Continuous smokers | 1711 | 78 | 7.84 | 1.83 (1.45, 2.30) |  |
| BMI<25 kg/m^2^ | Non-smokers | 61976 | 2254 | 6.16 | 1 (ref.) | 0.031 |
|  | New smokers | 3141 | 161 | 8.70 | 1.67 (1.41, 1.96) |  |
|  | Quitters | 4954 | 231 | 7.98 | 1.39 (1.21, 1.60) |  |
|  | Continuous smokers | 18712 | 967 | 8.88 | **1.85 (1.70, 2.02)** |  |
| BMI≥25 kg/m^2^ | Non-smokers | 64878 | 2313 | 5.98 | 1 (ref.) |  |
|  | New smokers | 3238 | 127 | 6.55 | 1.54 (1.28, 1.84) |  |
|  | Quitters | 5417 | 191 | 5.89 | 1.25 (1.07, 1.45) |  |
|  | Continuous smokers | 19275 | 713 | 6.21 | 1.63 (1.48, 1.79) |  |
| **All-cause Mortality** | | |  |  |  |  |
| < 65 years | Non-smokers | 88512 | 1444 | 2.69 | 1 (ref.) | 0.001 |
|  | New smokers | 5386 | 155 | 4.71 | 1.57 (1.32, 1.86) |  |
|  | Quitters | 8593 | 311 | 5.93 | 1.78 (1.56, 2.02) |  |
|  | Continuous smokers | 33635 | 1162 | 5.72 | 1.92 (1.76, 2.10) |  |
| ≥ 65 years | Non-smokers | 38342 | 3364 | 14.84 | 1 (ref.) |  |
|  | New smokers | 993 | 153 | 26.78 | 1.42 (1.20, 1.67) |  |
|  | Quitters | 1778 | 329 | 32.55 | 1.63 (1.45, 1.83) |  |
|  | Continuous smokers | 4352 | 760 | 30.84 | 1.65 (1.52, 1.80) |  |
| Men | Non-smokers | 59010 | 2914 | 8.22 | 1 (ref.) | 0.120 |
|  | New smokers | 5792 | 281 | 7.99 | 1.42 (1.26, 1.61) |  |
|  | Quitters | 9410 | 593 | 10.44 | 1.62 (1.48, 1.78) |  |
|  | Continuous smokers | 36276 | 1864 | 8.56 | 1.74 (1.64, 1.86) |  |
| Women | Non-smokers | 67844 | 1894 | 4.62 | 1 (ref.) |  |
|  | New smokers | 587 | 27 | 7.77 | 1.89 (1.29, 2.77) |  |
|  | Quitters | 961 | 47 | 8.20 | **2.21 (1.65, 2.95)** |  |
|  | Continuous smokers | 1711 | 58 | 5.71 | 1.75 (1.34, 2.29) |  |
| BMI<25 kg/m^2^ | Non-smokers | 61976 | 2947 | 7.93 | 1 (ref.) | 0.081 |
|  | New smokers | 3141 | 216 | 11.41 | 1.51 (1.31, 1.74) |  |
|  | Quitters | 4954 | 430 | 14.57 | 1.69 (1.52, 1.87) |  |
|  | Continuous smokers | 18712 | 1331 | 11.96 | 1.75 (1.63, 1.89) |  |
| BMI≥25 kg/m^2^ | Non-smokers | 64878 | 1861 | 4.74 | 1 (ref.) |  |
|  | New smokers | 3238 | 92 | 4.67 | 1.35 (1.09, 1.67) |  |
|  | Quitters | 5417 | 210 | 6.36 | 1.62 (1.40, 1.88) |  |
|  | Continuous smokers | 19275 | 591 | 5.07 | 1.74 (1.57, 1.93) |  |

*Adjusted for age, sex, body mass index, income status, hypertension, dyslipidemia, fasting blood glucose, kidney function, use of insulin, number of oral hypoglycemic agents, alcohol intake and exercise status

**Table S4**. Hazard ratios (HRs) and 95% confidence intervals of cardiovascular disease (CVD) and all-cause mortality according to change of exercise or smoking status: Sensitivity analysis excluding subjects with the occurrence of end points within 1 year of follow-up

|  | Number of individuals (n) | Number of events (n) | Incidence rate  (per 1000 person-years) | Model 1 | Model 2 |
| --- | --- | --- | --- | --- | --- |
| **CVD** (myocardial infarction or stroke) | | |  |  |  |
| Nonexercisers | 118723 | 4221 | 7.17 | 1 (ref.) | 1 (ref.) |
| New exercisers | 27778 | 786 | 5.65 | 0.81 (0.75, 0.87) | 0.83 (0.77, 0.90) |
| Exercise dropouts | 19051 | 666 | 7.02 | 0.88 (0.81, 0.96) | 0.91 (0.83, 0.98) |
| Constant exercisers | 14381 | 384 | 5.33 | 0.68 (0.61, 0.76) | 0.72 (0.65, 0.80) |
| **All-cause Mortality** |  |  |  |  |  |
| Nonexercisers | 118723 | 4578 | 7.65 | 1 (ref.) | 1 (ref.) |
| New exercisers | 27778 | 898 | 6.37 | 0.84 (0.78, 0.90) | 0.86 (0.80, 0.93) |
| Exercise dropouts | 19051 | 776 | 8.05 | 0.89 (0.83, 0.97) | 0.92 (0.85, 0.99) |
| Constant exercisers | 14381 | 474 | 6.51 | 0.72 (0.66, 0.80) | 0.76 (0.69, 0.84) |
| **CVD** (myocardial infarction or stroke) | |  |  |  |  |
| Nonsmokers | 125817 | 3975 | 6.34 | 1 (ref.) | 1 (ref.) |
| New smokers | 6309 | 248 | 7.86 | 1.58 (1.38, 1.80) | 1.59 (1.39, 1.81) |
| Quitters | 10219 | 350 | 6.85 | 1.28 (1.14, 1.43) | 1.27 (1.13, 1.42) |
| Continuous smokers | 37588 | 1484 | 7.98 | 1.79 (1.67, 1.91) | 1.78 (1.66, 1.91) |
| **All-cause Mortality** |  |  |  |  |  |
| Nonsmokers | 125817 | 4233 | 6.66 | 1 (ref.) | 1 (ref.) |
| New smokers | 6309 | 274 | 8.54 | 1.52 (1.34, 1.72) | 1.52 (1.34, 1.72) |
| Quitters | 10219 | 543 | 10.47 | 1.66 (1.51, 1.82) | 1.65 (1.50, 1.81) |
| Continuous smokers | 37588 | 1676 | 8.86 | 1.82 (1.71, 1.94) | 1.81 (1.69, 1.92) |

Model 1; adjusted for age, sex, body mass index, income status, hypertension, dyslipidemia, fasting blood glucose, kidney function, use of insulin, and number of oral hypoglycemic agents

Model 2; model 1 + adjusted for alcohol intake and exercise or smoking status

**Table S5.** Hazard ratios (HRs) and 95% confidence intervals of cardiovascular disease (CVD) and all-cause mortality according to change of exercise or smoking status: Sensitivity analysis excluding subjects with a history of cancer at baseline

|  | Number of individuals (n) | Number of events (n) | Incidence rate  (per 1000 person-years) | Model 1 | Model 2 |
| --- | --- | --- | --- | --- | --- |
| **CVD** |  |  |  |  |  |
| Nonexercisers | 104641 | 4057 | 6.52 | 1 (ref.) | 1 (ref.) |
| New exercisers | 24224 | 745 | 5.12 | 0.80 (0.74, 0.87) | 0.83 (0.77, 0.90) |
| Exercise dropouts | 16509 | 622 | 6.30 | 0.87 (0.80, 0.95) | 0.89 (0.82, 0.97) |
| Constant exercisers | 12440 | 349 | 4.67 | 0.65 (0.59, 0.73) | 0.69 (0.62, 0.77) |
| **All-cause Mortality** |  |  |  |  |  |
| Nonexercisers | 104641 | 3788 | 5.99 | 1 (ref.) | 1 (ref.) |
| New exercisers | 24224 | 731 | 4.95 | 0.83 (0.77, 0.90) | 0.86 (0.80, 0.94) |
| Exercise dropouts | 16509 | 638 | 6.36 | 0.90 (0.825, 0.98) | 0.92 (0.85, 1.01) |
| Constant exercisers | 12440 | 407 | 5.39 | 0.76 (0.69, 0.85) | 0.81 (0.73, 0.89) |
| **CVD** | |  |  |  |  |
| Nonsmokers | 108870 | 3727 | 5.73 | 1 (ref.) | 1 (ref.) |
| New smokers | 5663 | 248 | 7.33 | 1.64 (1.44, 1.87) | 1.66 (1.45, 1.89) |
| Quitters | 8849 | 342 | 6.44 | 1.35 (1.21, 1.52) | 1.35 (1.20, 1.51) |
| Continuous smokers | 34432 | 1456 | 7.14 | 1.77 (1.65, 1.89) | 1.78 (1.66, 1.90) |
| **All-cause Mortality** |  |  |  |  |  |
| Nonsmokers | 108870 | 3398 | 5.15 | 1 (ref.) | 1 (ref.) |
| New smokers | 5663 | 231 | 6.70 | 1.57 (1.37, 1.79) | 1.56 (1.36, 1.78) |
| Quitters | 8849 | 418 | 7.74 | 1.69 (1.52, 1.88) | 1.68 (1.51, 1.87) |
| Continuous smokers | 34432 | 1517 | 7.30 | 1.96 (1.83, 2.10) | 1.93 (1.80, 2.07) |

Model 1; adjusted for age, sex, body mass index, income status, hypertension, dyslipidemia, fasting blood glucose, kidney function, use of insulin, and number of oral hypoglycemic agents

Model 2; model 1 + adjusted for alcohol intake and exercise or smoking status

**Figure S1**. Study design and categorization of the study population.

Health examination at the time of DM diagnosis (1^st^ exam): new-onset type 2 DM

Health examination after DM diagnosis (2^nd^ exam)

2-years interval

| Groups categorized by physical activity status before and after DM diagnosis | | | |
| --- | --- | --- | --- |
| No | 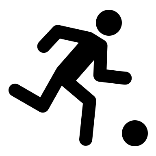 | No | Nonexercisers |
| No |  | Yes | New exercisers |
| Yes |  | No | Exercise dropouts |
| Yes |  | Yes | Constant exercisers |
| Groups categorized by smoking status before and after DM diagnosis | | | |
| No | 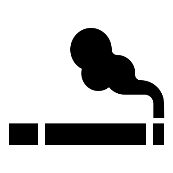 | No | Nonsmokers |
| No |  | Yes | New smokers |
| Yes |  | No | Quitters |
| Yes |  | Yes | Continuous smokers |
